# Mental health problems in the 10^th^ grade and non-completion of upper secondary school: the mediating role of grades in a population-based longitudinal study

**DOI:** 10.1186/1471-2458-14-16

**Published:** 2014-01-09

**Authors:** Åse Sagatun, Sonja Heyerdahl, Tore Wentzel-Larsen, Lars Lien

**Affiliations:** 1 Centre for Child and Adolescent Mental Health, Eastern and Southern Norway, Oslo, Norway; 2 Norwegian Centre for Violence and Traumatic Stress Studies, Oslo, Norway; 3 National Centre for Dual Diagnosis, Innlandet Hospital Trust, Sanderud, Brumundal, Norway; 4 Faculty of Public Health, Hedmark University College, Elverum, Norway

## Abstract

**Background:**

School drop-out is a problem all over the world with adverse life-course consequences. The aim of this paper is to study how internalising and externalising problems in the 10^th^ grade are associated with non-completion of upper secondary school, and to examine the mediating role of grade points in the 10^th^ grade across general academic and vocational tracks in upper secondary school. We also study the impact of health behaviour.

**Methods:**

Population-based health surveys were linked with Norwegian registries on education and sociodemographic factors (n = 10 931). Mental health was assessed by the self-report Strengths and Difficulties Questionnaire. Logistic regression was used to analyse the relations between mental health and health behaviour in 10^th^ grade and non-completion of upper secondary school. The mediating effect of grade points was studied by causal mediation analysis.

**Results:**

Adolescents not completing upper secondary school reported more externalising problems and girls more internalising problems in the 10^th^ grade, after adjustments. Smoking and physical inactivity increased the odds of non-completion of upper secondary school. Causal mediation analyses showed that a reduction in externalising problems of 10 percentage points led to lower rates of non-completion of 4–5 percentage points, and about three-quarters of this total effect was mediated by grades. For internalising problems the total effect was significant only for girls (1 percentage point), and the mediated effect of grades was about 30%. The effect of mental health problems on school dropout was mainly the same in both vocational and general tracks.

**Conclusions:**

Assuming a causal relationship from mental health problems to school performance, this study suggests that externalising problems impair educational attainment. A reduction of such problems may improve school performance, reduce school drop-out and reduce the adverse life-course consequences.

## Background

School drop-out is a national problem in countries all over the world and has an impact on both the individual and the society [1]. Lack of education has adverse life-course consequences, such as poor health and greater demand on social welfare entitlements. Understanding why students drop out of school is a key to designing effective interventions to reduce this critical and costly problem. Drop-out of school is likely to be influenced by an array of factors. These factors may be related to characteristics and experiences of the students themselves, including health, behaviour and performance, as well as characteristics and features of their environment, such as families and friends, schools and communities [2]. Mental health problems, including emotional, behavioural and peer-related problems, are prevalent among adolescents and may severely interfere with everyday functioning [3,4]. Some population-based studies have found that both hyperactivity and conduct problems [5-7] and depression [7-11] are negatively associated with subsequent educational attainment. A few population based studies from the USA and New Zealand have also accounted for co-occurring disorders [12-14] and found that especially externalising problems impair educational attainment. School performance is an important predictor for drop-out [2], and symptoms of mental health problems may undermine performance and represent a pathway for an effect of mental health on educational attainment. Different mental health problems may affect educational outcome through distinct causal pathways and may therefore require separate approaches to interventions. Studies’ elaborating on possible mechanisms for the associations between various mental health problems and educational outcome are hard to find. Different educational tracks such as general/academic or vocational education and training may require unique skills, and mental health problems may have different consequences for educational attainment in academic and non-academic tracks. To our knowledge, this has not previously been studied. When considering a causal connection of mental health problems on educational attainment, it is important to point out that these factors could be linked with other causes contributing to the association, creating a spurious effect. The risk literature for externalising and internalising problems and academic attainment provides clues about potential common causes, such as sociodemographic factors and health behaviour including physical activity, smoking and alcohol habits [13,15,16].

### The present study

We study the extent to which mental health problems, both internalising (emotional and peer problems) and externalising problems (conduct problems, hyperactivity–inattention), in the 10^th^ grade (age 15–16 years) are associated with non-completion of upper secondary school during the following 5 years. Furthermore, we study the mediating role of grade points when analysing the effect of mental health problems in the 10^th^ grade on non-completion of upper secondary school in a causal modelling framework. Finally, we investigate whether the patterns detected are the same in general and vocational tracks in upper secondary school. In addition to adjusting for family background and demographic factors, we study the impact of health behaviour. Analyses are stratified by gender.

## Methods

### Study population and procedure

This paper is based on a large and comprehensive cross-sectional survey of all 10^th^ graders (age 15–16 years) in six Norwegian counties, which was linked to the high-quality Norwegian registries for education, income and country of birth (of participants and their parents). The Norwegian Institute of Public Health conducted these comprehensive health surveys between 2000 and 2004. The students completed two four-page questionnaires during two school classes. A project assistant was present in the classroom to inform the students about the survey and to administer the questionnaires. For those not present on the day of the survey, questionnaires, informed consent forms and a pre-stamped envelope were left for them at school. Students who did not return the completed questionnaire during the course of the school year were prompted by a letter sent to their home. All parents received written information, and the students signed an informed consent form at baseline indicating their acceptance of the linkage of survey data to registry data. Permission to use the survey data was given by The Norwegian Institute of Public Health and the study was approved by the Regional Committee for Medical and Health Research Ethics and by the Norwegian Data Inspectorate.

The overall response rate of the questionnaire studies was 87% (*n* = 12 434). Of the participants in the questionnaire studies, 88% (*n* = 10 931) accepted linkage of information between the survey and official registers. The 12 percent that did not accept linkage to the national registers did not differ significantly in gender distribution compared to our study sample (*p* = 0.782). The two groups did not differ significantly in symptoms of internalising (*p* = 0.581) or externalising problems (*p* = 0.164).

### Measures

#### Non-completion of upper secondary school and grade points

Information on completion of upper secondary school and the final grade points in the 10^th^ grade were provided by Statistics Norway. In accordance with Statistics Norway, we define non-completers as individuals not completing upper secondary education within 5 years after ending the 10^th^ grade. To classify participants into general and vocational tracks, we used the track first entered after the 10^th^ grade (description of the Norwegian school system below). The final grades (0–6) in the 11 main school subjects from 10^th^ grade are summed to the variable “grade points” (0–66). To pass a course, a grade of 2 or higher must be achieved.

#### The Norwegian school system

The Norwegian school system is by and large public. The right to free public education is available for 13 years, of which 10 are compulsory. After completing compulsory education to the 10^th^ grade at 15–16 years of age, about 98% in Norway continue into post-compulsory/upper secondary school [17]. There are no general entry requirements, apart from having completed the 10^th^ grade, but there are entry requirements to certain educational programmes. If there are more applicants than the number of vacant places, admission to a programme depends on grade points from the 10^th^ grade. Upper secondary education consists of both general and vocational tracks. The students in general tracks follow a 3-year line. After finishing and passing all exams, they are qualified to enter higher education. In the vocational programme, there are three paths of 3 or 4 years. Two of these lead to a practical occupation without qualification for higher education, and most students follow one of these two tracks. In the third option, it is possible to take courses that qualify for higher education in addition to the vocational programme.

#### Mental health problems

Mental health problems were assessed by the Strengths and Difficulties Questionnaire (SDQ), a multi-informant wide-angle screening questionnaire [18,19]. The SDQ has been used in a large number of population-based studies in several countries [19]. In the current study, the self-report version of the questionnaire was used. The SDQ is a 25-item questionnaire with five subscales, each consisting of five items, generating scores for emotional symptoms, conduct problems, hyperactivity–inattention, peer problems and prosocial behaviour. Each item can be answered with “not true” (0), “somewhat true” (1) or “certainly true” (2), with reference to the past 6 months. For each subscale these values were summed to generate scale scores ranging from 0 to 10 [19]. There is theoretical and empirical support for combining the SDQ’s emotional symptoms and peer problems subscales into an ‘internalising’ subscale, covering anxiety, depression and withdrawal. The conduct problems and hyperactivity-inattention subscales are combined into an ‘externalising’ subscale. In the current paper, we used the internalising and the externalising subscales with a possible range of scores from 0 to 20 with higher scores representing more problems [20]. Cronbach’s alpha was 0.69 for the internalising subscale and 0.68 for the externalising subscale. A difference of about 10 points on a 0–100 scale is often considered noticeable [21]. On a 0–20 scale, this corresponds to a difference of 2, which is the difference we use when presenting the regression and mediation analyses.

#### Health behaviour

##### Smoking habits

Were studied by asking ‘Do you smoke, or have you smoked earlier?’ Possible answers were: ‘No, never’, ‘Yes, but I have stopped’, ‘Yes, once in a while’ and ‘Yes, daily’. The answers were categorised into ‘never/quit’, ‘once in a while’ and ‘daily’.

##### Alcohol consumption

Was estimated by asking how often in the course of the past year the person had been drinking alcohol. Answers were coded into six categories: ‘not at all’, ‘a few times’, ‘once per month’, ‘2–3 times per month’, ‘once per week’ and ‘2–3 times per week or more’.

##### Physical activity

Includes various types of activities in leisure time, and both organised and unorganised activities. The measure takes into account the amount of both moderate and vigorous physical activity. Participants were asked how many hours per week they spent on physical activity ‘to an extent that makes you sweat and/or out of breath’ outside of school. The possible answers were: 0, 1–2, 3–4, 5–7, 8–10, or 11 hours or more per week.

#### Sociodemographic factors and family background

Participants were from the north and south-east regions of Norway. The northern counties are Troms (the study was conducted the school year 2002/2003), Finnmark (2002/2003) and Nordland (2003/2004), and the south-eastern counties are Oppland (2001/2002), Hedmark (2000/2001) and Oslo (2000/2001).

Statistics Norway’s definition of *ethnic minorities*, as those having both parents born in a country other than Norway, was applied [22]. Information about parents’ country of birth was provided by register data from Statistics Norway. About 13% of the study population had an ethnic minority background. *Mother’s and father’s education* at the participants’ age 15–16 was used to identify ‘parents’ education’. The highest accomplished education of the parents was used, and information was provided by Statistics Norway’s register on education. In the analyses, parents’ education was categorised as ‘compulsory education’, ‘intermediate education’, ‘tertiary education’ and ‘tertiary education, more than 4 years’ [23].

##### Mother’s and father’s income

At the participants’ age 15–16 was used and provided by Statistics Norway’s register on income. The mother’s and father’s income [24] was categorised as ‘high’ (above the 75^th^ percentile), ‘medium’ (25^th^ to 75^th^ percentile), or ‘low’ (below the 25^th^ percentile) in the descriptive table. In multiple regression analyses, income was entered as a continuous variable, with winsorisation of values above 2.5 million NOK (replacing values above this income by 2.5 million NOK, this is about 450 000 USD) because of a highly skewed distribution.

##### Parents’ marital status

Was registered by a question asking whether parents were ‘married/cohabitant’, ‘unmarried’, ‘divorced/separated’, ‘one or both dead’ and ‘other options’. The participants were also asked whom they lived with (i.e., mum and dad; mum or dad). These two questions were combined to give information about parents’ marital status and whom the students lived with at the age of 15–16 years. The participants were classified into those living with both parents or having parents who were married/cohabitants, versus those living with mum or dad or others.

#### Statistics

All analyses were stratified by gender. To describe the distribution of the background factors and health behaviour, cross-tab analysis was performed. The latter was also used to describe the non-completion rates in these strata. To compare the SDQ scores and grade points among those completing and not completing upper secondary school, and those following vocational and general tracks, we used *t* tests. To study the correlation between health behaviour and mental health, we used Spearman’s rank order correlation.

To examine the extent to which variations in symptoms of mental health problems and health behaviour in the 10^th^ grade were associated with non-completion of upper secondary school, we performed logistic regression analyses. First we studied the effect of internalising and externalising problems separately (Model 1). In order to study the independent effect of externalising problems we adjusted for internalising scores and vice versa (Model 2). In the final model we also included health behaviours (Model 3). To examine the homogeneous odds ratio (OR) assumption for the internalising and externalising scales, the logistic regressions were re-run with these variables modelled by restricted cubic splines with four knots [25].

Causal mediation analysis [26,27], with upper secondary grade points as mediator, was used to study the direct and indirect effects of internalising and externalising problems on non-completion of upper secondary school (Additional file 1: Figure S1, online). Indirect effects are the effects mediated via grades, and the direct effects are the effects not mediated. For model identification, the causal mediation analysis makes the untestable sequential ignorability assumption, which in particular implies that there are no unmeasured confounders [27]. Briefly, sequential ignorability means that [27, page 310] conditional on the confounders, actual treatment (corresponding to internalising, resp. externalising problems in our case) is independent of all potential values of outcome and mediator, and, also conditioning on actual treatment, the actual mediator is independent of all potential values of the mediator. Potential here refers to a counterfactual setting. Since this assumption is untestable sensitivity analysis is recommended. Specifically [27, Theorem 2 page 316], model identification is also possible under deviations from sequential ignorability, assuming a specific correlation *ρ* between the error terms in the models for the mediator and the outcome, with zero correlation corresponding to sequential ignorability. Such an error correlation is reasonable when there are unmeasured confounders for outcome and mediator. When the direct and indirect effects have the same sign for a large *ρ* interval, the sensitivity of the conclusions of the causal mediation analysis is considered as small. Direct and indirect effects were studied for a 2-point increase in SDQ score, from 4 to 6 for internalising problems and from 6 to 8 for externalising problems; these intervals were chosen within the main range of the respective distributions. The effect measure is increase in drop-out probability. The analyses were repeated for another 2-point interval within the main ranges to check for possible sensitivity of conclusion to the choice of interval. No substantial differences were found. Sensitivity analysis was performed as described above. Because of present limitations in the software, the sensitivity analyses were based on a probit instead of a logit model for drop-out, and we checked whether this changed the causal mediation analysis appreciably. To determine whether the relationships between mental health and non-completion of upper secondary school were the same in both general and vocational tracks, we re-ran the logistic regressions and causal mediation analyses for the two tracks separately. We created 95% confidence intervals for the ratio by bootstrapping. In the mediation analyses, differences between tracks were tested by an approximate procedure inferring standard errors based on the estimated effects and differences between effects and confidence bounds.

In the descriptive analyses and the logistic regression, SPSS 18 (SPSS Inc., Chicago, IL, USA) was used. Other analyses used R version 2.15.2 (The R Foundation for Statistical Computing, Vienna, Austria), with the R packages *mediation (4.1.2) *[26] for causal mediation analysis and *boot* for bootstrapping.

## Results

### Background characteristics

Of the study population, 32.4% did not complete upper secondary education within 5 years after finishing compulsory school (including 2% not starting upper secondary school). More boys (38.9%) than girls (25.8%) were non-completers. The percentage starting at vocational tracks was 52.8 among boys and 41.0 among girls. For both genders, the prevalence of not completing upper secondary school was about three times higher for vocational tracks (Table 1). Background characteristics are presented in Table 1.

**Table 1:** Background characteristics and rate of non-completion of upper secondary school (n = 10931)

	**Boys (5511)**		**Girls (5420)**	
	**Proportion of population**	**Non-completion rate percentage**	**Proportion of population**	**Non-completion rate percentage**
Sample dropout rate	50.4	38.9	49.6	25.8
General tracks	47.2	19.4	59.0	12.6
Vocational traks	52.8	53.8	41.0	38.8
**Sociodemographic factors**				
Ethnic background^a^	(n = 5511)		(n = 5419)	
Ethnic Norwegian	90.0	37.3	90.2	24.9
1st-generation immigrants	6.4	57.5	6.4	37.9
2st-generation immigrants	3.6	45.0	3.4	25.1
County of residence in Norway	(n = 5511)		(n = 5420)	
Oslo	31.8	33.8	31.3	21.4
Hedmark	16.2	43.1	16.5	25.0
Oppland	16.2	32.8	15.9	22.0
Nordland	19.4	41.8	19.1	32.0
Troms	11.1	44.1	12.5	28.2
Finnmark	5.2	53.3	5.2	37.0
**Family factors**				
Parents’ education at age 16	(n = 5438)		(n = 5367)	
Compulsory	13.6	62.6	13.4	46.2
Intermediate	44.0	44.7	44.5	30.8
Tertiary	30.8	28.3	29.8	15.4
Tertiary more than 4 years	11.6	14.9	12.3	8.8
Fathers’ income at age 16	(n = 5291)		(n = 5112)	
Low	34.3	50.2	32.6	33.8
Medium	34.6	35.8	35.9	24.9
High	31.1	26.3	31.5	15.5
Mothers’ income at age 16	(n = 5412)		(n = 5334)	
Low	32.1	45.5	32.6	31.7
Medium	34.3	40.1	34.6	27.8
High	33.6	30.2	33.1	17.0
Parents’ marital status/who do you live with	(n = 5395)		(n = 5369)	
Mum and dad married/cohabiting	67.5	33.0	66.5	19.5
Mum or dad or other	32.5	49.3	33.5	37.7

^a^See definition in text.

The final grade points in 10^th^ grade were significantly higher among those who completed upper secondary school than those who did not complete it [(mean (SD): boys 45.5 (7.2) vs. 35.6 (7.6); girls 48.7 (6.8) vs. 38.3 (7.8); all *p <* 0.001)]. Mental health problems were also negatively associated with grades [(Pearson correlation, *r*): SDQ externalising, boys (−0.38, *p* < 0.001), girls (−0.41, *p* < 0.001) and SDQ internalising, boys (−0.13, *p* < 0.001), girls (−0.24, *p* < 0.001)].

### The association between mental health problems in the 10^th^ grade, health behaviour in the 10^th^ grade and non-completion of upper secondary school

Boys and girls who did not complete upper secondary school reported more internalising and externalising problems in the 10^th^ grade than those who completed upper secondary school, independent of track in upper secondary school (Table 2). Those following vocational tracks reported more mental health problems than those following general tracks, independent of gender.

**Table 2:** Strengths and Difficulties Questionnaire (SDQ) in the 10th grade by gender, type of track and completion of upper secondary school after 5 years

	**Boys**								**Girls**							
**Mental health**	**Completers (3348)**				**Non-completers (2082)**				**Completers (4000)**				**Non-completers (1380)**			
	**Mean**	**SD**	**(95% CI)**		**Mean**	**SD**	**(95% CI)**		**Mean**	**SD**	**(95% CI)**		**Mean**	**SD**	**(95% CI)**	
**General tracks**																
SDQ externalising	4.91	2.96	4.79	5.04	6.45	3.07	6.17	6.72*	5.04	2.64	4.94	5.14	6.81	3.15	6.49	7.14*
SDQ internalising	3.08	2.56	2.97	3.19	3.72	2.90	3.46	3.98*	4.34	2.94	4.22	4.45	5.39	3.27	5.05	5.73*
**Vocational tracks**																
SDQ externalising	5.99	3.04	5.82	6.15	7.29	3.26	7.13	7.46*	6.03	2.82	5.88	6.18	7.58	3.11	7.38	7.78*
SDQ internalising	3.40	2.57	3.26	3.54	3.87	2.93	3.73	4.02*	5.15	3.12	4.99	5.31	6.06	3.35	5.84	6.28*

*Statistically significant difference between completers and non-completers, *p* ≤ *0.001.*

Adolescents who were smokers and used alcohol frequently had a higher non-completion rate than those who used these substances less. Those physically active outside of school had a lower non-completion rate. These patterns were independent of gender (Tables 3, 4 and 5, crude analysis). The correlations between the mental health scores, between the health behaviours and mental health, and between health behaviours were all 0.40 or below for both genders, except the correlation between smoking and alcohol use among girls (*r* = 0.49).

**Table 3:** **Health behaviour in the 10**^**th **^**grade according to non-completion of upper secondary school (n = 10931)**

	**Boys (5511)**		**Girls (5420)**	
	**Proportion of population**	**Non-completion rate percentage**	**Proportion of population**	**Non-completion rate percentage**
**Health behaviour in the 10**^**th **^**grade**				
Smoking	(n = 5474)	*	(n = 5394)	*
No	74.2	32.5	66.8	19.0
Sometimes	12.4	44.4	16.3	25.9
Yes, every day	13.4	68.9	16.9	52.4
Alcohol last year	(n = 5387)	*	(n = 5337)	*
Never	26.5	35.7	22.3	23.5
A few times last year	27.2	30.7	30.2	19.7
1–3 times a month	32.1	42.1	34.5	28.9
Once a week or more	14.2	52.2	13.1	35.2
Physical activity	(n = 5390)	*	(n = 5248)	*
0 hour per week	10.4	50.5	12.3	37.1
1–2 hours per week	19.4	45.7	29.6	31.1
3–4 hours per week	20.8	37.9	27.1	22.1
5–7 hours per week	23.1	34.0	20.4	16.4
8 hours or more per week	26.4	32.3	10.7	18.8

* Statistically significant difference between completer and non-completers, *p* ≤ *0.001.*

**Table 4:** **Mental health problems and health behaviour in the 10**^**th **^**grade as predictors of non-completion of upper secondary school in boys (n = 4760)**

	**Crude**			**Model 1**^**a**^			**Model 2**^**b**^			**Model 3**^**c**^		
	**OR**	**95% CI**		**OR**	**95% CI**		**OR**	**95% CI**		**OR**	**95% CI**	
**SDQ externalising (per 2 points)**	1.43	1.38	1.49*	1.38	1.32	1.44*	1.38	1.32	1.45*	1.27	1.21	1.33*
**SDQ internalising (per 2 points)**	1.17	1.12	1.22*	1.11	1.06	1.16*	1.00	0.95	1.05	1.01	0.96	1.06
**Health behaviour**												
Smoking												
No	ref.		*							ref.		*
Sometimes	1.82	1.53	2.17							1.56	1.27	1.91
Yes, every day	4.83	4.02	5.80							2.80	2.25	3.48
Alcohol last year												
Never	ref.		*							ref.		^**d**^
A few times last year	0.91	0.77	1.08							0.88	0.72	1.06
1–3 times a month	1.41	1.21	1.66							1.02	0.84	1.24
Once a week or more	2.21	1.82	2.68							1.36	1.06	1.73
Physical activity												
0 hours per week	ref.		*							ref.		*
1–2 hours per week	0.82	0.66	1.03							0.92	0.71	1.18
3–4 hours per week	0.61	0.49	0.76							0.71	0.55	0.91
5–7 hours per week	0.53	0.43	0.66							0.72	0.56	0.92
8 hours or more per week	0.47	0.38	0.58							0.62	0.49	0.80

^a^Model 1: SDQ externalising and SDQ internalising separately, adjusted for ethnic background, county of residence, parents’ education, income and merital status.

^b^Model 2: SDQ externalising and SDQ internalising adjusted for each other, and for ethnic background, county of residence, parents’ education, income and merital status.

^c^Model 3: Like model 2, in addition adjusted for health behaviours.

*p ≤ 0.001.

^**d**^*p* = 0.003.

**Table 5:** **Mental health problems and health behaviour in the10**^**th **^**grade as predictors of non-completion of upper secondary school in girls (n = 4751)**

	**Crude**			**Model 1**^**a**^			**Model 2**^**b**^			**Model 3**^**c**^		
	**OR**	**95% CI**		**OR**	**95% CI**		**OR**	**95% CI**		**OR**	**95% CI**	
**SDQ externalising (per 2 points)**	1.59	1.51	1.66*	1.48	1.41	1.56*	1.44	1.36	1.52*	1.31	1.24	1.39*
**SDQ internalising (per 2 points)**	1.29	1.24	1.35*	1.21	1.16	1.27*	1.08	1.03	1.14*	1.09	1.04	1.15*
**Health behaviour**												
Smoking												
No	ref.		*							ref.		*
Sometimes	1.46	1.21	1.77							1.30	1.05	1.62
Yes, every day	4.75	4.01	5.62							2.77	2.23	3.45
Alcohol last year												
Never	ref.		*							ref.		
A few times last year	0.85	0.70	1.04							0.83	0.66	1.05
1–3 times a month	1.36	1.13	1.64							0.88	0.69	1.13
Once a week or more	1.92	1.53	2.41							0.93	0.68	1.26
Physical activity												
0 hours per week	ref.		*							ref.		*
1–2 hours per week	0.77	0.63	0.95							1.12	0.89	1.43
3–4 hours per week	0.49	0.39	0.61							0.85	0.66	1.10
5–7 hours per week	0.34	0.27	0.44							0.65	0.49	0.86
8 hours or more per week	0.41	0.31	0.54							0.78	0.57	1.07

^a^Model 1: SDQ externalising and SDQ internalising separately, adjusted for ethnic background, county of residence, parents’ education, income and marital status.

^b^Model 2: SDQ externalising and SDQ internalising adjusted for each other, and for ethnic background, county of residence, parents’ education, income and marital status.

^c^Model 3: Like model 2, in addition adjusted for health behaviours.

*p ≤ 0.001.

When adjusting for sociodemographic factors and family background in the logistic regression (Tables 4 and 5, Model 1), the ORs for both externalising and internalising problems were slightly reduced. When including both the SDQ subscales in the same model, together with sociodemographic factors and family background (Model 2), the OR for non-completion of upper secondary school predicted by a 2-point increase in externalising problems remained mainly the same as in Model 1 in both genders. In this model (Model 2) the ORs for internalising problems were reduced; in boys the OR was statistically insignificant [1.00 (0.95–1.05)] and in girls 1.08 (1.03–1.14). In the final model, also including health behaviours (Model 3), the ORs for non-completion of upper secondary school predicted by a 2-point increase in internalising symptoms remained mainly the same as in the previous model in both genders. The ORs for externalising problems in this model were reduced in both genders, but still significant [boys; 1.27 (1.21–1.33) and girls; 1.31 (1.24–1.39)].

To test whether the presented OR values were representative for the whole scale, we tested the heterogeneity of OR. We found no heterogeneity of ORs across the internalising scale for girls (*p* for non-linearity = 0.950) or boys (*p =* 0.777), or for the externalising scale among girls (*p =* 0.563). However, there was a statistically significant heterogeneity in externalising problems for boys (*p =* 0.009). Specifically, the ORs per 2-point increase in externalising problems were somewhat higher for low values of the externalising problems score. In the range comprising most of the data (Additional file 2: Figure S2, online), the ORs were close to 1.27, and in the sequel, we use a linear relationship also for externalising problems for boys.

In the final model (Model 3), smoking, alcohol use and physical activity remained independent predictors for non-completion of upper secondary school among the boys (Table 4, Model 3). In girls, smoking and physical activity remained independent predictors (Table 5, Model 3).

### The mediating role of grade points when analysing the effect of mental health in 10^th^ grade on non-completion of upper secondary school, and the impact of health behaviour

In the causal mediation analysis, we found that a 2-point decrease in externalising problems in the proposed model would result in a 6.9 percentage point reduction in the probability of non-completion of upper secondary school in boys, after adjustment for internalising problems, family background and sociodemographic factors (Table 6, Model 2). Of this total effect, 71.4% was mediated by grade points. In girls, the corresponding figures were 6.3 and 73.2% (Table 6, Model 2). When adjusting for health behaviour (Model 3), the total effect of a 2-point reduction in externalising problems would lead to a reduction of 4.7 percentage points in boys and 4.4 percentage points in girls in the probability of non-completion of upper secondary school. Of these effects, 83.1% and 75.5% were mediated by grade points among boys and girls, respectively. Among girls, a 2-point decrease in internalising problems gave a total reduction in the probability of non-completion of upper secondary school of 1.2 percentage points. Of this total effect, 41.2% was mediated by grade points. After adding health behaviour, the corresponding figures were 1.2 and 31.0%. In boys, internalising problems did not significantly affect the non-completion of upper secondary school via grade points in the 10^th^ grade.

**Table 6:** The effect of a 2-point change in the SDQ subscales on non-completion of upper secondary school, and the mediating effect of grades

	**Model 1**^**a**^						**Model 2**^**b**^						**Model 3**^**c**^					
	**Total effect**			**Proportion via mediation**			**Total effect**			**Proportion via mediation**			**Total effect**			**Proportion via mediation**		
	**Estimate**	**95% CI**		**Estimate**	**95% CI**		**Estimate**	**95% CI**		**Estimate**	**95% CI**		**Estimate**	**95% CI**		**Estimate**	**95% CI**	
**Boys**																		
SDQ externalising	0.068	0.059	0.077	0.702	0.611	0.801	0.069	0.060	0.079	0.714	0.629	0.813	0.047	0.038	0.058	0.831	0.692	1.032
SDQ internalising	0.021	0.010	0.031	0.540	0.324	0.938*	−0.00056	−0.010	0.010	0.428	−12.90	14.00	0.0031	−0.006	0.013	−0.740	−14.20	22.90*
**Girls**																		
SDQ externalising	0.067	0.057	0.077	0.716	0.628	0.833*	0.063	0.053	0.073	0.732	0.624	0.860*	0.044	0.033	0.055	0.755	0.610	0.976*
SDQ internalising	0.031	0.023	0.039	0.622	0.488	0.823*	0.012	0.005	0.020	0.412	0.185	0.952*	0.012	0.005	0.020	0.310	0.108	0.687*

^a^Model 1: Adjusted for ethnic background, county of residence, parents education, income and marital status.

^b^Model 2: SDQ externalising and SDQ internalising adjusted for each other, and for ethnic background, county of residence, parents education, income and marital status.

^c^Model 3: Like model 2, in addition adjusted for physical activity, smoke and alcohol habits.

*Direct effect statistically significant (p < 0.05).

The total effect estimate is the change in the probability of non-completion.

The sensitivity analyses (Additional file 3: Figure S3a and b, online) showed that, as long as *ρ* is above −0.4, the indirect effects still had the same sign as estimated, indicating acceptable robustness of these findings. For direct effects of internalising problems, the signs were as indicated for *ρ* between −0.7 and 0.8, indicating strong robustness. However, the sensitivity analysis for direct effects of externalising problems showed low robustness (*ρ* < 0.2 for girls and *ρ* < 0.1 for boys). There were only minor differences in the causal mediation analyses using a logit or probit model for drop-out.

### The importance of general and vocational tracks

When studying the association between mental health in the 10^th^ grade and non-completion of upper secondary school in logistic regression by general and vocational tracks, no significantly different patterns were detected (95% CI for the ratio crossed one). The mediation analysis, stratified by general and vocational tracks (Model 3), revealed similar patterns for the effect of mental health problems on non-completion in both tracks, except when studying internalising problems in girls. Here the total effect of internalising problems was stronger in vocational (2 percentage points) than in general tracks (0.1 percentage points) (*p* difference = 0.019), with a greater proportion mediated by grades (*p* difference = 0.018).

## Discussion

### Summary of main results

Of the 10^th^ graders participating in the study, around one-third did not complete upper secondary school within the following 5 years. Both boys and girls who did not complete school reported more externalising problems, and girls more internalising problems in the 10^th^ grade after adjustments for background factors, health behaviour and co-occurring symptoms of mental health problems. Smoking and physical inactivity were also significantly associated with higher odds of not completing upper secondary school in the final model for both boys and girls. The causal mediation analyses suggested that a reduction in externalising problems of 2 points lead to lower rates of non-completion in upper secondary school by 4–5 percentage points for both genders. About three-quarters of this total effect was mediated by grades. For internalising problems, the total effect was lower and significant only for girls. The effect of mental health problems on non-completion of upper secondary school was mainly the same in general and vocational tracks.

### Methodological strengths and limitations

The main strengths of the study include the use of a representative sample of young people in schools in different counties in Norway, the availability of national registers for grades, education and SES variables, and the longitudinal nature of the data that made prospective analysis possible. Assessments of both internalising and externalising problems alongside information on educational achievement at follow-up were used. To our knowledge, there are no prospective European studies analysing both internalising and externalising problems in a representative sample of youth. Novel statistical methods are used to estimate possible causal mediation pathways of non-completion of upper secondary school [26,27]. A limitation of the study is that not all those invited took part in the baseline study, and not all participants accepted the survey to be linked with national registers. Thus our analyses are based on 77% of all 10^th^ graders in the respective counties for the years in question. Of the participants in the baseline study 12 percent did not accept linkage of their survey. Even though the participants not accepting linkage did not differ significantly in their report of symptoms of mental health problems we might have a selective loss to follow-up. However the percentage in our study not completing upper secondary school within 5 years is in accordance with Statistics Norway’s national numbers for the comparable years (68%–72%) [17]. There is also some empirical support that generalisation for a measure of association is less sensitive to loss to follow-up than prevalence measures [28]. A paper based on parts of the same sample considers response rates and selection problems by investigating mental health and health behaviour variables [28]. Here the association measures (prevalence ratios) were quite similar among participants and all invitees. The response rate was quite high, and we expect that the findings are fairly representative for the study population. Another limitation is that mental health problems are only assessed by self-report. However, in a study of the psychometric properties of the SDQ, the self-report problem scales: emotional symptoms, conduct problems and hyperactivity/inattention were all found to be associated with the relevant DSM-IV diagnoses (based on interviews) [29]. The OR for having a psychiatric disorder in high (above the 90th percentile) rather than low risk groups was 9.7 for the emotional symptoms scale in relation to emotional disorder; 7.1 for the conduct problems scale in relation to oppositional defiant disorder or conduct disorder and 5.0 for the hyperactivity/inattention scale in relation to ADHD disorders [29], supporting the validity of the self-report scale. The self-report version of the SDQ total has also shown satisfactory discrimination between community and clinical samples [30]. Another methodological aspect that has to be taken into consideration when interpreting the results from the causal mediation analysis is that we estimated direct and indirect effects of mental health on the probability of non-completion of upper secondary school based on a theoretical model. The reliability of the results relies on the trustworthiness of the model. We have included several background factors that previously have been shown to be associated with the exposure, mediator and outcome. Still our model, like most models, simplifies complex mechanisms, and we cannot rule out any residual confounding. Based on previous literature, we believe that important confounders are included in the model. In sensitivity analyses, our results seem to be robust for moderate residual correlations, corresponding to a moderate amount of unmeasured confounders in the models for the mediator and outcome. Nevertheless, early conduct problems have been found to be associated with lower IQ and attention difficulties, with the result that the difficulties faced by children with conduct problems were exacerbated by lower average IQ and higher rates of attentional problems [31]. Our measure of externalising problems includes symptoms of both conduct problems and hyperactivity–inattention problems, but we have not measured IQ. Other comparable studies have included IQ and still find an effect of externalising problems on educational attainment at follow-up (see later) [12,14].

We found a weak to moderate correlation between externalising and internalising problems, but a causal relationship cannot be excluded. However, we have studied the two variables both separately and controlled for each other. In the causal mediation analyses, the estimates for externalising problems were quite similar in the models without versus with adjustment for internalising problems, while the estimates related to the internalising scale became weaker when adjusting for externalising problems (Model 1 and 2). We cannot rule out that we might have underestimated the effect of internalising symptoms by controlling for externalising problems. Another debatable issue associated with our model is whether health behaviour should be adjusted for (Model 3), as health behaviour may be influenced by mental health. We presented results both without and with adjustment for health behaviour (Models 2 and 3) and found that by including health behaviours, the effect of externalising problems on non-completion of upper secondary school decreased. By controlling for health behaviour, we may have over-adjusted in Model 3 and under-estimated the effect. Another issue is that we tested to what degree mental health in the 10^th^ grade affects non-completion of upper secondary school through grade points. It is also plausible that the effect is the opposite; i.e., that years of struggle with school and low grades might have led to disengagement and feeling of worthlessness and poor mental health before our baseline study.

### Our results according to previous findings, and possible explanations

#### Mental health and subsequent educational achievement

When considering externalising and internalising problems separately, we found that both problem scales were significant predictors for non-completion of upper secondary school. However, when considering both externalising and internalising problems together, we found that externalising problems was a stronger predictor than internalising problems. Other studies that have focused on the effect of internalising [7-11] or externalising problems/disorders [5-7,31] on subsequent educational achievements report that both problem spectra are significantly associated with educational outcome. In one study including both internalising and externalising disorders in the same analysis Breslau and colleagues [13] used a US national sample and studied with a retrospective design the joint effect of childhood and adolescent onset of psychiatric disorders on failure to graduate from high school on time. After adjusting for smoking, alcohol use, use of illegal drugs, prior disorders, childhood adversity, and sociodemographic characteristics (including gender), significant associations with failure to graduate on time remained for conduct disorder and the three ADHD subtypes. Internalising disorders were not significantly associated with failure to graduate on time after these adjustments. Miech and colleagues [14] followed a New Zealand cohort from birth to the age of 21 (The Dunedin Study), and studied mental disorders at age 15 and failure to earn ‘a sixth form certificate’, which is comparable with the high school degree in the USA and upper secondary school in Norway. The internalising disorders of anxiety and depression, whether assessed by DSM diagnoses or DSM symptom scales, did not significantly affect educational attainment. In contrast, externalising disorders (conduct disorders and attention deficit disorders) impaired educational achievement. The analyses were adjusted for socioeconomic background, gender, IQ, reading ability, school involvement and the presences of other mental disorders. Another longitudinal study following a normative urban school sample of 205 children from the USA over 20 years found that externalising problems evident in childhood appeared to undermine academic competence by adolescence [12]. This effect did not differ by gender and persisted after controlling for IQ, parenting quality and socioeconomic differences. Our findings in the logistic regression are in line with these findings from the USA and New Zealand. We stratified by gender and found that internalising problems were a significant predictor for non-completion among girls but not among boys.

We also studied the mediated effect through grades for the effect of internalising and externalising problems on non-completion of upper secondary school. A large proportion of the total effect of externalising problems was mediated by grades in both genders. The total effect of internalising problems was only significant in girls, and the direct effect constituted the greatest part of the total effect. We have not found other longitudinal studies performing such analyses, nor have we found studies comparing different educational tracks when studying the mediating role of grades. That grades play an important role in dropping out from school is well-known [2]. Less studied is whether the role of grades as a mediator of the effects of externalising and internalising problems on non-completion of upper secondary school differs. Nevertheless, the findings are plausible when considering the nature of the problems. Externalising problems (conduct problems and hyperactivity–inattention) have a more stable nature and may reflect a cumulative effect of inattention and learning across the schooling career, which may have an impact on the acquisition of academic skills. Conduct disorders lead to repeated disciplinary actions, which are likely to affect students’ engagement with schooling and to influence their grades. Internalising problems (symptoms of depression, anxiety and peer problems) are likely to disrupt students’ overall social functioning and perceived competence, leading to diminished motivation, but may have a shorter term nature, and may be less associated with academic skills. It is important to note that there is evidence for adverse consequences of internalising disorders on other outcomes, such as establishing themselves in the job market, and establishment of friendship and romantic relationships [4].

We found that boys and girls starting vocational tracks reported more mental health problems in the 10^th^ grade and had poorer grade points and a higher non-completion rate in upper secondary school than those starting general tracks. However, the associations (OR) between mental health problems and non-completion of upper secondary school, and the mediating effects of grades, were similar, indicating that the mechanisms are comparable within the two types of educational tracks.

#### Health behaviour and subsequent educational achievement

We found that smoking predicted non-completion of upper secondary school in both genders, after adjustments for symptoms of mental health problems, other health behaviours, family factors and sociodemographic background. This finding is in line with the study by Breslau and colleagues [13]. They highlight the distinct role of tobacco use as a predictor of high school graduation. Breslau and colleagues also included use of alcohol and illegal drugs, and substance misuse disorders in their study. After including tobacco use, together with childhood adversity and sociodemographic characteristics, substance use disorders were no longer associated with high school graduation. The strong effect of smoking/tobacco in our study might be due to a common factor not controlled for, such as genetic influences or negative peer relations. It may also be that tobacco influences the brain in ways of which we are not fully aware.

In our study, physical activity outside of school in the 10^th^ grade was associated with lower odds of not finishing upper secondary school compared with no physical activity outside of school. Cross-sectional associations between physical inactivity and school difficulties such as grade repetition, low school performance and school drop-out ideation have previously been reported [15]. Physical activity is shown to influence physical health, exerting a powerful positive influence on the brain, and consequently on emotional stability and cognition, self-efficacy and motivation [32]. Based on our findings, we cannot draw a conclusion regarding causal mechanisms, but the findings are encouraging concerning ‘the battle’ of arguing for more physical education in the curriculum.

## Conclusions

Our results indicate that externalising problems in particular impair completion of upper secondary school. Externalising problems impair educational attainment, with a negative influence on future life chances. Treatment and preventive interventions targeted at these adolescents are of special importance. A Canadian study found that early preventive intervention for those at high risk of antisocial behaviour increased the rates of high-school completion [33]. Reduction of externalising problems may improve later school performance, reduce school drop-out, and reduce the risk of poor health, exclusion from the labour market and dependency on welfare entitlements. Our findings encourage interventions to prevent externalising problems and to promote healthy behaviour among youth as a tool to reduce non-completion of upper secondary school.

## Competing interests

The authors declare that they have no competing interests.

## Authors’ contributions

ÅS was active in the planning of the study, did the conception and design of the paper, analysed and interpreted the data and drafted the manuscript. SH was involved in the conception and design of the paper, discussed the analysis and interpretation of the data, and reviewed the manuscript critically. TWL participated in the conception and design of the paper, did the statistical analyses together with the first author, and reviewed the manuscript critically. LL was involved in the conception and design of the paper, discussed the analysis and interpretation of the data, and reviewed the manuscript critically. All authors read and approved the final manuscript.

## Pre-publication history

The pre-publication history for this paper can be accessed here:

http://www.biomedcentral.com/1471-2458/14/16/prepub

## Supplementary Material

Additional file 1: Figure S1 The model assumed in the causal mediation analyses. Click here for file

Additional file 2: Figure S2 (A) Odds ratio (OR) by 2 points less for externalising problems, with 95% confidence interval in boys. There is a statistical significant inhomogeneity of OR (*P* = 0.009). The dotted line is an average OR of 1.27, assuming linearity. (B) Histogram for the distribution of the externalising problem scores in boys. Click here for file

Additional file 3: Figure S3 a. Graphical display of Results from the sensitivity analyses for direct and indirect effect of externalising problems by gender (‘Medsens’ function). Results as a function of ρ. b. Graphical display of results from the sensitivity analyses for direct and indirect effect of internalising problems by gender (‘Medsens’ function). Results as a function of ρ. Click here for file
