# Supplementary figures and images for: Mental health problems in the 10th grade and non-completion of upper secondary school: the mediating role of grades in a population-based longitudinal study
Source: BMC Public Health. 2014 Jan 9;14:16. doi: 10.1186/1471-2458-14-16 (PMC3905670; doi:10.1186/1471-2458-14-16)

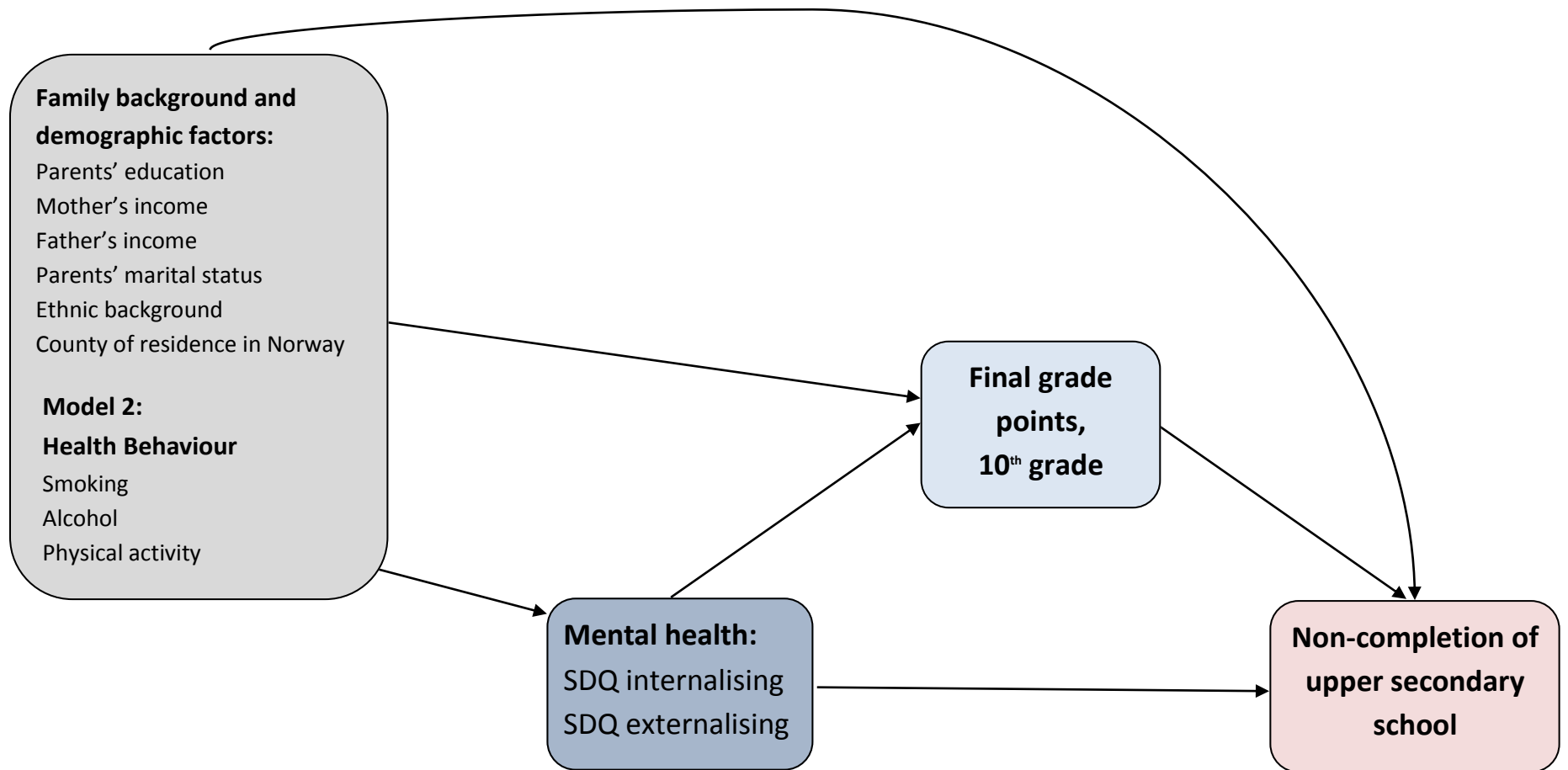

**Additional file 1: Figure S1-** The model assumed in the causal mediation analyses.

Supplement: Additional file 1: Figure S1 — The model assumed in the causal mediation analyses. [file 1471-2458-14-16-S1.pdf]
