# Supplementary material for: Mental health problems in the 10th grade and non-completion of upper secondary school: the mediating role of grades in a population-based longitudinal study
Source: BMC Public Health. 2014 Jan 9;14:16. doi: 10.1186/1471-2458-14-16 (PMC3905670; doi:10.1186/1471-2458-14-16)
Supplement: Additional file 2: Figure S2 — (A) Odds ratio (OR) by 2 points less for externalising problems, with 95% confidence interval in boys. There is a statistical significant inhomogeneity of OR (P = 0.009). The dotted line is an average OR of 1.27, assuming linearity. (B) Histogram for the distribution of the externalising problem scores in boys. [file 1471-2458-14-16-S2.pdf]

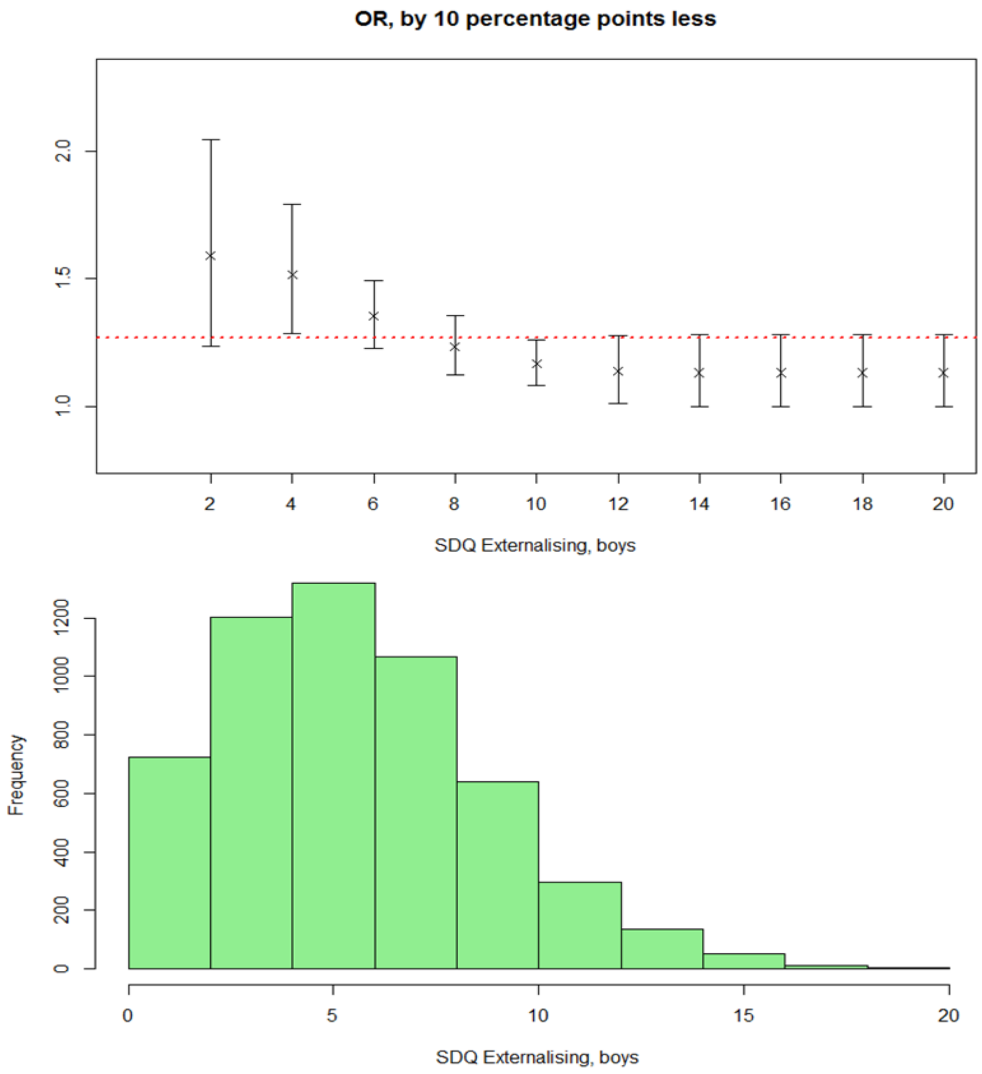

Additional file 2: Figure S2- (A) Odds ratio (OR) by 2 points less for externalising problems, with 95% confidence interval in boys.

There is a statistical significant inhomogeneity of OR (*P* = 0.009). The dotted line is an average OR of 1.27, assuming linearity.

(B) Histogram for the distribution of the externalising problem scores in boys.
