# Supplementary material for: Mental health problems in the 10th grade and non-completion of upper secondary school: the mediating role of grades in a population-based longitudinal study
Source: BMC Public Health. 2014 Jan 9;14:16. doi: 10.1186/1471-2458-14-16 (PMC3905670; doi:10.1186/1471-2458-14-16)
Supplement: Additional file 3: Figure S3 — a. Graphical display of Results from the sensitivity analyses for direct and indirect effect of externalising problems by gender (‘Medsens’ function). Results as a function of ρ. b. Graphical display of results from the sensitivity analyses for direct and indirect effect of internalising problems by gender (‘Medsens’ function). Results as a function of ρ. [file 1471-2458-14-16-S3.pdf]

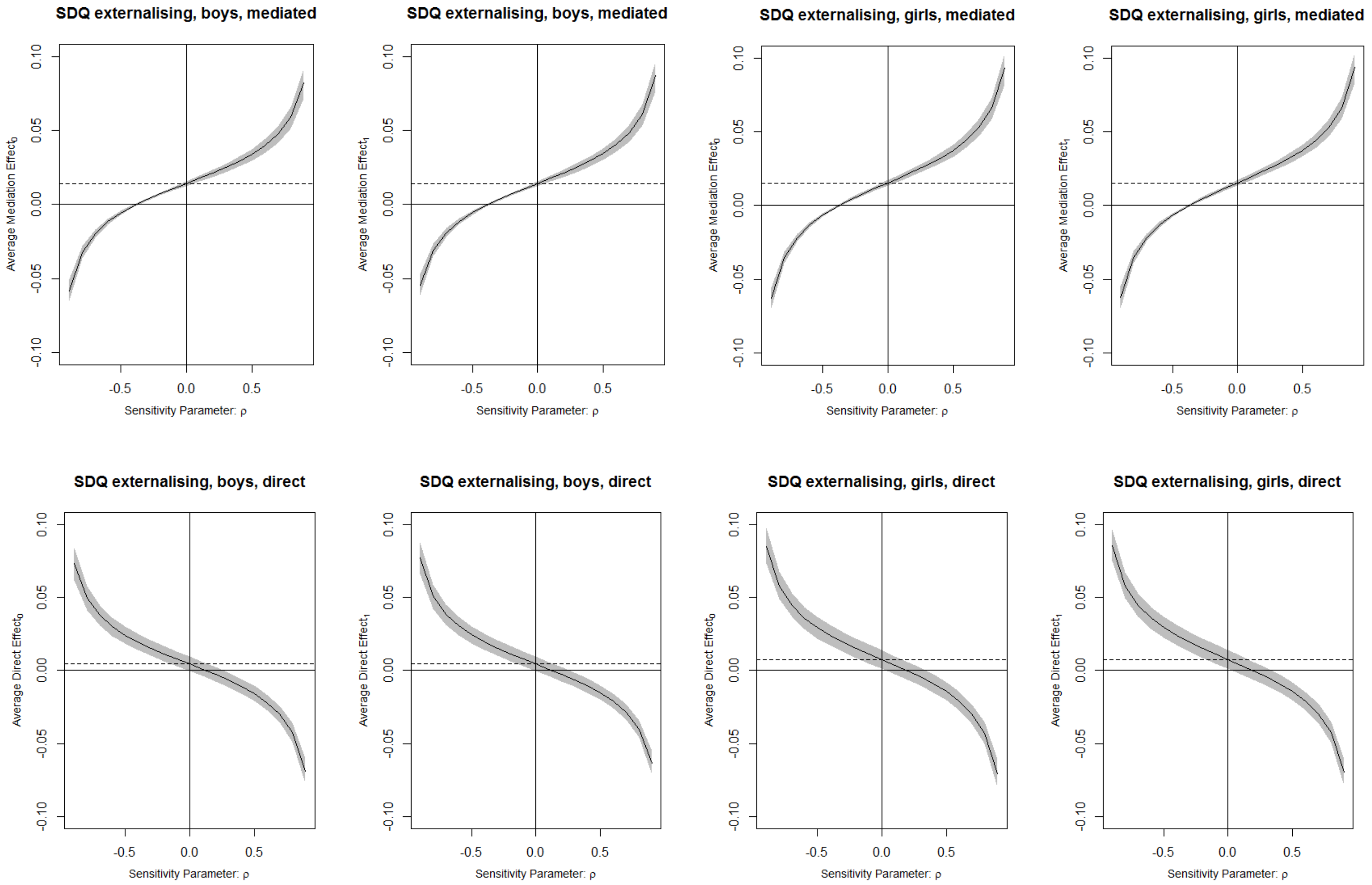

Additional file 3: Figure S3a - Graphical display of Results from the sensitivity analyses for direct and indirect effect of externalising problems by gender (‘Medsens’ function). Results as a function of ρ.

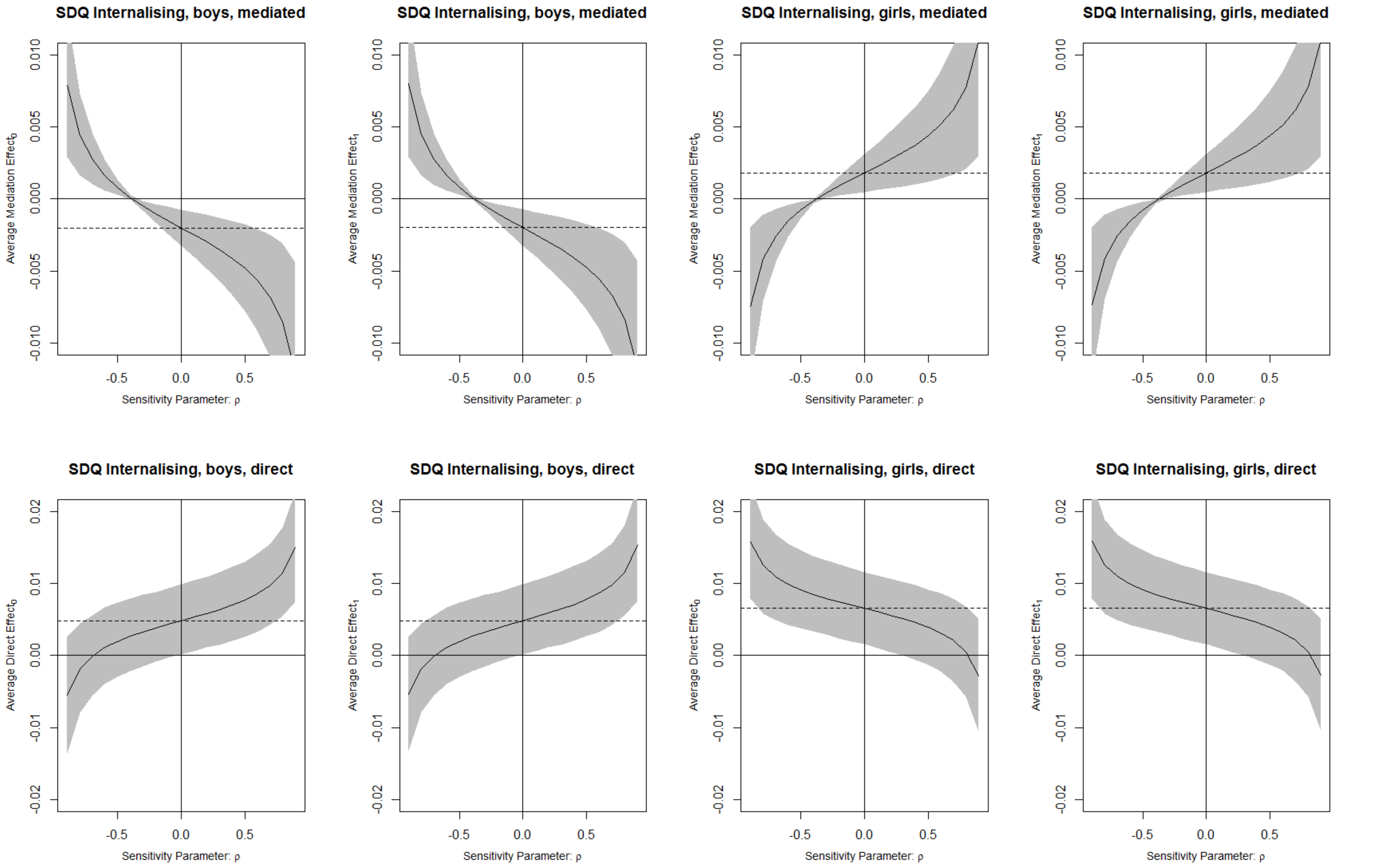

Additional file 3b - Graphical display of results from the sensitivity analyses for direct and indirect effect of internalising problems by gender ('Medsens' function). Results as a function of ρ.
